# The effectiveness of short-term pulmonary rehabilitation program in patients with comorbid asthma, chronic obstructive pulmonary disease and obesity

**DOI:** 10.25122/jml-2021-0050

**Published:** 2022-02

**Authors:** Olha Huivaniuk, Hanna Stupnytska, Oleksandr Fediv, Andriy Bocharov

**Affiliations:** 1. Department of Propaedeutic of Internal Diseases, Bukovinian State Medical University, Chernivtsi, Ukraine; 2. Department of Internal Medicine and Infectious Diseases, Bukovinian State Medical University, Chernivtsi, Ukraine; 3. Department of Surgery No.1, Bukovinian State Medical University, Chernivtsi, Ukraine

**Keywords:** BODE-index, ACO, obesity, PR – pulmonary rehabilitation, ACO – asthma-COPD overlap, COPD – chronic obstructive pulmonary disease, BMI – Body mass index, CAT – COPD Assessment Test, ACT – Asthma Control Test, mMRS – modified Medical Research Council dyspnea scale, 6MWT – six-minute walking test, BODE index – body mass index, forced expiratory volume in one second, dyspnoea, and 6-min walk distance, ICS – inhaled corticosteroid, GINA – Global Initiative for Asthma, GOLD – Global Initiative for Obstructive Lung Disease, FEV1 – forced expiratory volume in 1 second

## Abstract

The effectiveness of pulmonary rehabilitation (PR) has not yet been established in patients with asthma – chronic obstructive pulmonary disease overlap (ACO) depending on their nutritional status. We aimed to evaluate the effectiveness of a short-term PR program in patients with comorbid asthma, chronic obstructive pulmonary disease (COPD), and obesity. We included 40 ACO patients and divided them into 3 groups according to body mass index (BMI) and then subdivided them into PR (n=21) and control (n=19) groups. The COPD Assessment Test (CAT), the Asthma Control Test (ACT), and the modified Medical Research Council dyspnea scale (mMRS) were used to evaluate symptoms levels. BODE index (body mass index, forced expiratory volume in one second, dyspnoea, and 6-min walk distance) was used to evaluate the effectiveness of pulmonary rehabilitation. In addition, spirometry and bioimpedansometry were performed. All measurements were done before and after a 6-month PR program. A significantly lower decline in the BODE index was observed in overweight patients (decreased by 43.6% compared to baseline and lower by 40.7% compared to the control group). The six-minute walking test (6MWT) significantly increased in all groups (p<0.001). There was a decrease in total CAT score by 25.4% and by 31.2% in the overweight group (p<0.001). The BMI decreased more in the obese group (by 9.4% compared to baseline). Our study result showed that early use of PR program significantly improves functional capacity and BODE index, leads to dyspnea and CAT scores reduction and improvement in pulmonary function, cause a decrease in BMI, body fat percentage, and visceral fat level, and an increase in muscle mass in overweight and obese patients with ACO.

## Introduction

There has been increasing recognition of the clinical overlap between asthma and chronic obstructive pulmonary disease (COPD) in recent years, known as asthma-COPD overlap (ACO). Patients with ACO have more respiratory symptoms, more frequent exacerbations and hospital admissions, lower quality of life, a more noticeable decline in lung function, and a higher mortality rate than those with asthma or COPD alone [1–4]. Currently, special attention is being paid to various clinical manifestations of asthma and COPD, along with markedly different patterns of chronic inflammation of the airways and lung parenchyma, and important comorbidities that contribute to the severity of the disease in an individual patient. Obesity is one of the most common factors leading to pulmonary dysfunction, causing injury to ventilation mechanics and being related to systemic inflammation [5, 6]. Physical inactivity and obesity are linked to low-grade systemic inflammation, which may contribute to the inflammatory processes seen in many chronic diseases [7]. Pulmonary rehabilitation (PR) is widely accepted as the most effective non-pharmacologic management for chronic respiratory conditions. Many studies have demonstrated its effectiveness in reducing symptoms of dyspnoea and fatigue, increasing exercise capacity, and improving health-related quality of life [1, 8–11]. A PR program is associated with decreased dyspnea perception, anxiety, depression, and increased exercise tolerance in patients with uncontrolled asthma [11] and COPD [12]. However, the effectiveness of PR has not yet been proved in patients with ACO, depending on their nutritional status. The aim of the research was to evaluate the effectiveness of short-term pulmonary rehabilitation program in patients with comorbid asthma, COPD, and obesity.

## Material and Methods

A total of 40 ACO patients (mean age 64.86±9.81 years) were recruited into the study, and following baseline assessment were divided into three groups: normal body mass index (BMI 18.5–24.9) – n=13, overweight (BMI<25–29.9) – n=13, and obese patients (BMI≥30) n=14. Patients in all 3 groups were additionally subdivided into the PR group, which included 7 patients from each group (total n=21). The effectiveness of PR was evaluated compared with the control group (n=19). There were no significant differences in baseline demographic and clinical characteristics between PR and control groups. All subjects included were previously prescribed long-acting bronchodilators in combination with inhaled corticosteroids (ICS). The study was conducted from October 15, 2018, until February 10, 2020. All patients were included at the time of hospital admission due to exacerbation and received written and oral information about the study and their right to withdraw. The inclusion criteria were as follows: diagnosis of asthma, COPD, age more than 40 years, and spirometry with persistent airflow limitation, not fully reversible, but with current or historical variability. ACO was defined as the presence of features shared with both diseases and a similar number of features for asthma and COPD, according to the Global Initiative for Asthma (GINA)/ the Global Initiative for Obstructive Lung Disease (GOLD) criteria [13].

The exclusion criteria were clinically significant orthopedic or cognitive impairment, any previous history of thoracic surgical intervention, history of myocardial infarction, unstable cardiovascular disease (*e.g.*, unstable angina, unstable arrhythmias), BMI<18.5, severe renal impairment. The PR program included combined strength and endurance training five times a week for 30 minutes, education, breathing techniques, psychological consulting, and dietetic support for obese patients. All patients were interviewed with a series of questionnaires, including the COPD Assessment Test (CAT), the Asthma Control Test (ACT), and the modified Medical Research Council dyspnoea scale (mMRS) for evaluation of the symptom level. Spirometry testing was performed using a computer spirograph “BTL-08 SpiroPro” (Great Britain). To evaluate the effectiveness of PR, the BODE index (body mass index, forced expiratory volume in one second, dyspnoea, and 6-min walking test (6MWT) (performed in accordance with the American Thoracic Society/European Respiratory Society statement) was used. Weight and body composition variables were measured using a segmental body composition monitor (BC-601 Tanita, Japan). All measurements were done before and after a 6-month PR program. Data are presented as mean±standard deviation. The Mann-Whitney U test was used to compare differences between normal weight and obesity groups, normal weight and overweight groups, and overweight and obesity groups. Wilcoxon signed-rank test was used to compare the outcome variables in the PR and control groups at baseline and after six months. All statistical analyses were performed using SPSS Statistics 23.0.

## Results

**Figure 1.**
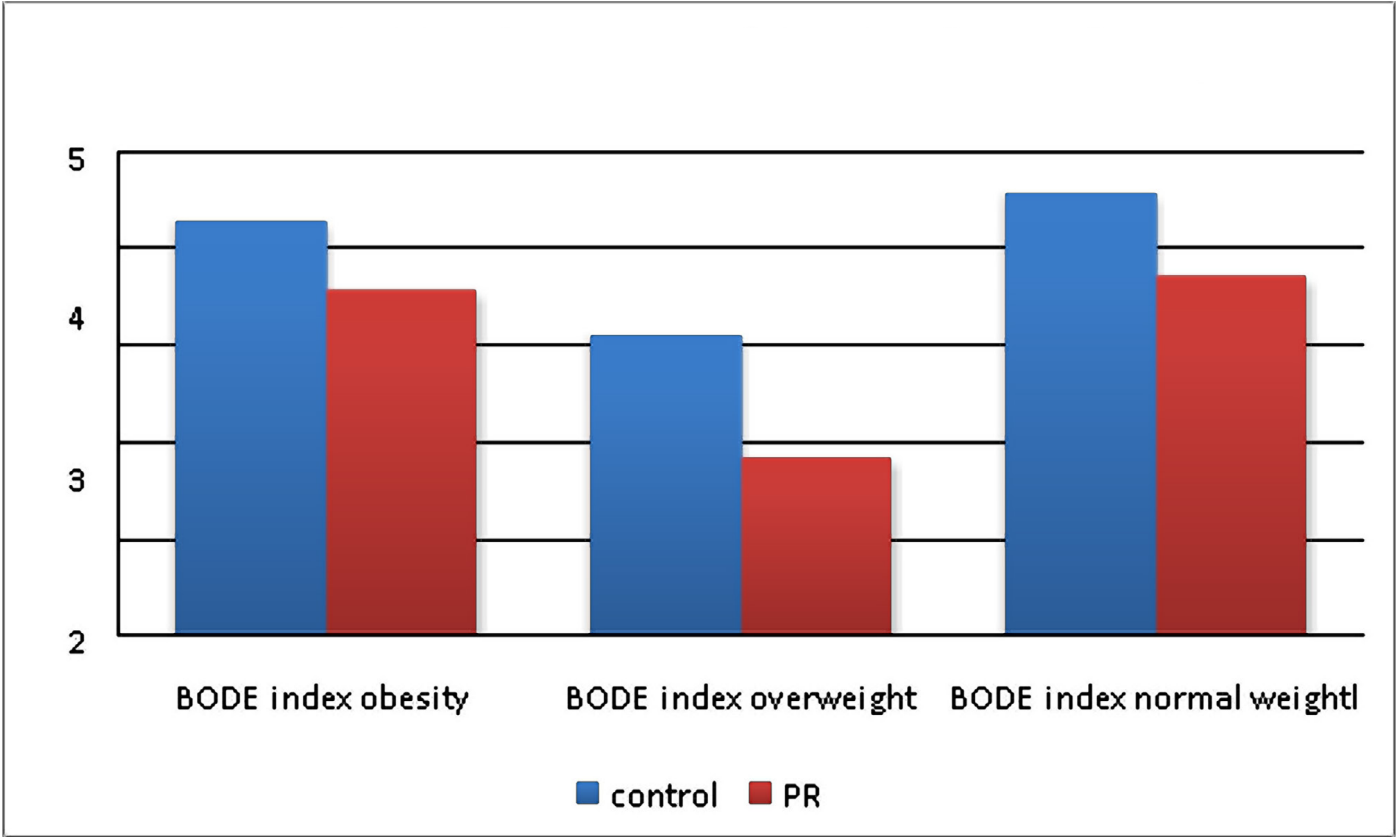
BODE index at 6 months in PR and control groups.

Baseline forced expiratory volume in 1 second (FEV1) was lower in patients with normal BMI by 5.4% compared to patients with obesity and 12.2% compared to overweight patients, but the difference was not significant (p>0.05) (Table 1). Overweight patients had better dyspnoea status (the lowest MRC dyspnoea scale scores) than patients in the obese and normal BMI groups. No significant difference was observed between the normal weight and overweight groups in ACQ-5, but the score was higher in patients with obesity. According to the 6MWT, patients’ exercise capacity was significantly lower in the obese group compared to other groups. The analysis of bioimpedansometry indicators demonstrated a significant difference in fat mass percentage between groups (p<0.001). There was a tendency towards lower muscle mass and a higher fat mass percentage in all groups. The BODE index was not significantly different between groups (Table 1). After six months, the BODE index decreased in each group reliably but was significantly lower in overweight patients (decreased by 43.6% compared to baseline and was lower by 40.7% than the control group) (Table 2, Figure1). There was a decrease in dyspnoea mMRC scale scores by 39.5% in the obese group and 45% in overweight patients (p=0.001). The 6MWT significantly increased in all groups (p<0.001). There was a decrease in total CAT score by 25.4% and 31.2% in the overweight group (p<0.001). The BMI was more decreased in the obese group (9.4% compared to baseline) than in the baseline group. The body fat percentage and visceral fat level decreased by 13.6% and 19.7%, respectively, in obese patients. FEV1 increased slightly after the PR program (by 5.1% in the overweight group, 6.1% in the obese group, and 4.2% in the normal BMI group) (Tables 2, 3 and 4).

**Table 1.** Baseline demographic and clinical characteristics of ACO patients with different nutritional status.

**Parameters**	**Obesity (BMI≥30) n=14**	**Overweight** **(BMI<25–29.9) n=13**	**Normal weight** **(BMI 18.5–24.9) n=13**	**p-value**
**Age, years**	57.07±4.53	55.5±3.41	56.75±4.22	>0.05
**BMI, kg/cm^2^**	34.04±0.68	27.9±0.69	20.5±1.87	p1<0.001; p2<0.001; p3<0.001
**Smoking, index of pack-years**	15.78±1.76	15±2.04	15.31±1.75	>0.001
**FEV1, % predicted**	63.32±12.27	68.19±10.58	59.92±9.02	>0.05
**mMRC, points**	2.55±0.85	2.07±0.99	2.73±0.59	>0.05
**CAT, points**	17.28±1.06	14.07±0.64	19.38±0.86	>0.05
**ACQ-5 score**	1.55±0.41	0.9±0.24	0.84±0.22	>0.05
**6MWT, m**	228.04±19.44	363.33±27.18	326±37.15	p1,p3<0.05; p2>0.05
**BODE index, points**	4.57±0.85	3.46±0.66	4.36±0.76	>0.05
**visceral fat level**	16.02±0.12	11.98±0.51	10.11±0.16	p1,p3<0.001; p2=0.002
**% fat mass**	30.59±1.29	27.71±2.30	18.87±2.09	<0.001
Muscle mass, kg	59.68±7.11	52.16±8.37	52.87±7.45	>0.001

p1 – difference between normal weight and obesity groups; p2 – difference between normal weight and overweight groups; p3 – difference between overweight and obesity groups.

**Table 2.** Outcomes for obese group before and after PR.

**Parameters**	**Baseline (n=7)**	**After 6-month (n=7)**	**Δ%**	**p-value**
**FEV1, % predicted**	63.08±11.12	66.9±10.88	+6.1	<0.001
**mMRC, points**	2.43±0.53	1.47±0.53	-39.5	=0.001
**CAT, points**	17.71±1.79	13.2±1.82	-25.4	<0.001
**ACQ-5 score**	1.51±0.5	0.88±0.25	-41.4	=0.003
**6MWT, m**	226.71±14.03	265.43±11.31	+17.07	<0.001
**BODE index**	4.71±0.75	3.57±0.97	-24.2	<0.001
**BMI, kg/cm^2^**	34.02±0.97	30.82±0.89	-9.4	<0.001
**visceral fat level**	16.2±0.3	13±0.41	-19.7	<0.001
**% fat mass**	30.18±1.08	26.07±1.09	-13.6	<0.001
**Muscle mass, kg**	58.06±12.7	59.33±12.6	+2.2	<0.001

**Table 3.** Outcomes for overweight group before and after PR.

**Parameters**	Baseline (n=7)	After 6-month (n=7)	Δ%	p-value
**FEV1, % predicted**	67.62±9.1	71.1±8.7	+5.1	<0.001
**mMRC, points**	2±0.8	1.1±0.69	-45	=0.001
**CAT, points**	13.71±0.5	9.43±0.78	-31.2	<0.001
**ACQ-5 score**	0.87±0.76	0.56±0.37	-35.8	=0.001
**6MWT, m**	363.33±27.18	416.71±13.02	+14.7	<0.001
**BODE index**	3.28±0.78	1.85±0.69	-43.6	=0.001
**BMI, kg/cm^2^**	27.85±0.67	26.2±0.85	-5.9	<0.05
**visceral fat level**	11.54±0.15	11.23±0.3		>0.05
**% fat mass**	27.03±1.76	25.32±1.09		>0.05
**Muscle mass, kg**	52.23±1.4	55.57±1.24	+6.4	<0.001

**Table 4.** Outcomes for normal weight group before and after PR.

**Parameters**	**Baseline (n=7)**	**After 6-month (n=7)**	**Δ%**	**p-value**
**FEV1, % predicted**	59.92±9.02	62.4±7.11	+4.2	>0.05
**mMRC, points**	2.73±0.59	2.1±0.34		>0.05
**CAT, points**	19.38±0.86	13.56±0.73	-30.0	=0.04
**ACQ-5 score**	0.82±0.42	0.68±0.25	-17.1	=0.003
**6MWT, m**	326±37.15	355.43±10.2		>0.05
**BODE index**	4.35±0.52	2.75±0.34	-36.7	=0.02
**BMI, kg/cm^2^**	20.7±1.56	20.02±1.04		>0.05
**visceral fat level**	9.87±0.17	9.53±0.25		>0.05
**% fat mass**	18.37±2.18	18.17±1.6		>0.05
**Muscle mass, kg**	53.03±8.1	52.65±7.76		>0.05

## Discussion

The coexistence of asthma and COPD features increases the burden of disease and challenges current diagnostic and therapeutic strategies, making the ACO a significant clinical entity. The GINA [1] and the GOLD [2] provided approaches for diagnosing and managing ACO; however, these recommendations are based mainly on expert opinion. PR is an important therapeutic approach for patients with respiratory diseases. A randomized controlled trial showed the effectiveness of a comprehensive six-week PR program in patients with ACOS (asthma-COPD overlap syndrome). It was reported that the 6-week PR program significantly improved functional capacity and health-related quality of life and BODE index [5]. Our study lasted 6 months and demonstrated that the PR program was most effective in overweight and obese patients. In particular, in these groups, there was a reduction in dyspnoea, CAT scores, and improvement in pulmonary function, as well as a more prominent increase in exercise tolerance. We found a significant improvement in 6MWT after 6 months of the PR program, which agrees with the findings of previous investigations [12, 14]. Recent studies found no significant differences in exercise capacity and health-related quality of life after PR in patients with COPD with different nutritional statuses. This study also found that the 6MWT significantly increased after the PR program in all groups, but the improvement was more prominent in overweight and obese patients than individuals with normal BMI. Celli *et al.* suggested using the BODE index to assess the effectiveness of rehabilitation programs [15]. We found that participation in the PR program was associated with a decrease in BODE index score, especially for overweight patients (in this group, the BODE index was lower by 43.6% compared to the control group). So, we suggest that the BODE index serves as a criterion for evaluating the short-term PR program in ACO patients with different nutritional statuses. It is known that chronic respiratory diseases are often associated with muscle wasting [16]. Simultaneously, excess body weight is associated with low-grade systemic inflammation and mechanical alterations in the airways and lung parenchyma [17]. Consequently, it is also important to analyze the body composition, including body fat percentage, visceral fat, and muscle mass. After 6 months of the PR program, we observed a decrease in BMI, body fat percentage, visceral fat level, and an increase in muscle mass in overweight and obese patients. We suggested that higher muscle mass could lead to a reduction of airflow obstruction in these groups.

Therefore, the analysis of the obtained data confirmed the effectiveness of the PR program in patients with comorbid asthma, COPD, and obesity. However, key limitations in the current study were the small number of participants and that our study design did not allow us to assess the optimal duration of the PR program. Therefore, future research is needed, and it is important to examine the effect of long-term PR in the ACO population, especially in the precision medicine era.

## Conclusion

Early use of PR program significantly improves functional capacity and BODE index, leading to dyspnoea and CAT score reduction and improvement in pulmonary function, causing a decrease in BMI, body fat percentage, and visceral fat level and an increase in muscle mass in overweight and obese patients with ACO.

## Acknowledgments

The study is a fragment of the planned research work of the Department of Propedeutics of Internal Diseases of the Bukovinian State Medical University– Medicamentic correction of metabolic and immunological disorders, intensity of systemic inflammation and endothelial dysfunction in patients with comorbid cardiovascular and broncho obstructive diseases, musculoskeletal disorders and chronic dermatoses (state registration number 0120U101550).

### Conflict of interest

The authors declare no conflict of interest.

### Ethical approval

This study was approved by the Committee on Bioethics of Higher State Educational Establishment of Ukraine, Bukovinian State Medical University, (No 2/2019).

### Consent to participate

Written informed consent was obtained from the participants in the study.

### Authorship

OH and HS contributed to conceptualizing, OF and AB contributed to the methodology, OH contributed to writing the original draft, HS and OF contributed to editing the manuscript, OH and AB contributed to data collection, OH and HS contributed to data analysis.
